# Alleged Hospital Abuse in Bradford

**Published:** 1903-02-07

**Authors:** 


### EDITOR'S LETTER-BOX.

**[Our Correspondents are reminded that prolixity is a great bar to publication, and that brevity of style and conciseness of statement greatly facilitate early insertion.]**

### ALLEGED HOSPITAL ABUSE IN BRADFORD.

Mr. Herbert J. Jeffery, Hon. Sec. Bradford Children's Hospital, writes: My attention has been drawn to a paragraph in your paper, quoting some remarks recently made by Mr. Bentham, Chairman of the Bradford Board of Guardians, alleging neglect of investigation on the part of the authorities of the Bradford Children's Hospital, as to the circumstances of the parents of children admitted to the benefits of the institution, and in order to refute such I cannot do better than send you the following extract from the report submitted by the board of management to the annual meeting of the Governors just held: "Very many of the cases treated at the Hospital are received on the recommendation of the medical men in the town, who naturally satisfy themselves that the position of the families is such as to justify an appeal to the charity, but the bulk of the in-patients are drafted into the Hospital by the hon. medical staff from the out-patients' department, after careful examination and inquiry as to the circumstances of each family, and when such circumstances are found not to justify admission patients are invariably refused. A further inquiry is subsequently made by the house committee, and if deemed necessary other investigations are instituted in the case of all in-patients, and by these means the risk of abuse of the Charity is greatly diminished, and the Board is satisfied that little abuse, if any, prevails." I entirely acquit Mr. Bentham of any intention to misrepresent the case, but that he has done so is abundantly evident from the report.

